# Weaning from mechanical ventilation in the operating room: a systematic review

**DOI:** 10.1016/j.bja.2024.03.043

**Published:** 2024-05-29

**Authors:** Megan Abbott, Sergio M. Pereira, Noah Sanders, Martin Girard, Ashwin Sankar, Michael C. Sklar

**Affiliations:** 1 Temerty Faculty of Medicine, University of Toronto, Toronto, ON, Canada; 2 Keenan Research Centre for Biomedical Science, St Michael's Hospital, Toronto, ON, Canada; 3 Department of Anesthesiology and Pain Medicine, University of Toronto, Toronto, ON, Canada; 4 Department of Anesthesiology, Centre Hospitalier de l’Université de Montréal, Montreal, QC, Canada; 5 Division of Critical Care, Department of Medicine, Centre Hospitalier de l’Université de Montréal, Montreal, QC, Canada; 6 Department of Anesthesiology, Centre Hospitalier de l’Université de Montréal Research Center, Montreal, QC, Canada; 7 Interdepartmental Division of Critical Care Medicine, University of Toronto, Toronto, ON, Canada

**Keywords:** fraction of inspired oxygen, mechanical ventilation, positive end-expiratory pressure, postoperative pulmonary complications, postoperative pulmonary outcomes, pressure support ventilation, weaning from mechanical ventilation

## Abstract

**Background:**

Postoperative pulmonary complications (PPCs) are associated with postoperative mortality and prolonged hospital stay. Although intraoperative mechanical ventilation (MV) is a risk factor for PPCs, strategies addressing weaning from MV are understudied. In this systematic review, we evaluated weaning strategies and their effects on postoperative pulmonary outcomes.

**Methods:**

Our protocol was registered on PROSPERO (CRD42022379145). Eligible studies included randomised controlled trials and observational studies of adults weaned from MV in the operating room. Primary outcomes included atelectasis and oxygenation; secondary outcomes included lung volume changes and PPCs. Risk of bias was assessed using the Cochrane Risk of Bias (RoB2) tool, and quality of evidence with the GRADE framework.

**Results:**

Screening identified 14 randomised controlled trials including 1719 patients; seven studies were limited to the weaning phase and seven included interventions not restricted to the weaning phase. Strategies combining pressure support ventilation (PSV) with positive end-expiratory pressure (PEEP) and low fraction of inspired oxygen (FiO_2_) improved atelectasis, oxygenation, and lung volumes. Low FiO_2_ improved atelectasis and oxygenation but might not improve lung volumes. A fixed-PEEP strategy led to no improvement in oxygenation or atelectasis; however, individualised PEEP with low FiO_2_ improved oxygenation and might be associated with reduced PPCs. Half of included studies are of moderate or high risk of bias; the overall quality of evidence is low.

**Conclusions:**

There is limited research evaluating weaning from intraoperative MV. Based on low-quality evidence, PSV, individualised PEEP, and low FiO_2_ may be associated with reduced postoperative pulmonary outcomes.

**Systematic Review Protocol:**

PROSPERO (CRD42022379145).

**Editor's key points**

• Intraoperative mechanical ventilation is a risk factor for postoperative pulmonary complications after general anaesthesia and surgery, but the impact of strategies for weaning from mechanical ventilation are understudied.
• In this systematic review, the authors evaluated weaning strategies and their effects on postoperative pulmonary outcomes.
• Based on 14 identified studies, strategies combining pressure support ventilation with positive end-expiratory pressure and low fraction of inspired oxygen (FiO_2_) improved atelectasis, oxygenation, and lung volumes, but the quality of evidence was low.
• Additional studies focusing on weaning from mechanical ventilation are critical to support the potential benefits of such interventions in reducing postoperative pulmonary complications.

Postoperative pulmonary complications (PPCs) are a leading cause of increased mortality and morbidity in surgical patients, especially during the first postoperative week.^1^, ^2^, ^3^ Depending on severity, risk factors, and definitions, the incidence ranges between 6% and 40%.^3^ The development of even one mild PPC is significantly associated with increased ICU admission, early postoperative mortality, and prolonged length of stay in both hospital and ICU.^4^ Atelectasis, the most frequent PPC, also leads to significant morbidity^4^ and is associated with patient risk factors, general anaesthesia, mechanical ventilation, and type of surgery.^5^ Atelectasis worsens patient outcomes through a reduction in lung volumes and a decrease in partial pressure of oxygen in arterial blood ($Pa_{O_{2}}$) and in the ratio of partial pressure of oxygen in arterial blood to fraction of inspiratory oxygen concentration ($Pa_{O_{2}}$/FiO_2_).^6^^,^^7^ Ultimately, atelectasis is associated with an increased risk of mortality,^4^ and is the main cause of postoperative hypoxaemia leading to postoperative respiratory failure and the need for respiratory support ranging from noninvasive oxygen use to invasive mechanical ventilation.^8^ Finally, atelectasis is also considered a risk factor for development of other PPCs such as pneumonia and ventilator-induced lung injury.^5^

Numerous strategies have been studied to reduce atelectasis, and in turn PPCs, during induction and maintenance of anaesthesia for major surgery. These studies have given rise to interventions including higher (fixed or individualised) positive end-expiratory pressure (PEEP),^9^, ^10^, ^11^ low FiO_2_,^12^^,^^13^ and recruitment manoeuvres.^14^^,^^15^ Most of these interventions decrease intraoperative atelectasis but fail to improve clinically relevant postoperative outcomes.^16^, ^17^, ^18^ Interestingly, imaging studies suggest similar postoperative atelectasis after extubation regardless of the intraoperative mechanical ventilation strategy used.^16^ There is paucity of research addressing the weaning and extubation phases. Conditions that contribute to atelectasis formation such as a high FiO_2_ and low PEEP settings are often observed during emergence from anaesthesia. Thus, current approaches to weaning from mechanical ventilation could counteract intraoperative strategies, facilitating atelectasis and potentially increasing the incidence of PPCs.

Although most patients undergoing surgery under general anaesthesia are extubated in the operating room, there currently is no definitive standard for weaning of mechanical ventilation. Recommendations from an expert panel-based consensus published in 2019, addressing intraoperative lung-protective mechanical ventilation, generated only weak recommendations for the weaning and extubation phases. Suggested interventions, such as avoiding zero end-expiratory pressure and high inspiratory FiO_2_ during the emergence phases, were based on limited evidence.^19^ In light of these limitations, we completed a systematic review to evaluate studies that proposed different weaning strategies from mechanical ventilation in the operating room and understand their effects on postoperative pulmonary outcomes.

## Methods

### Design

We conducted a systematic review with predetermined selection and outcome criteria. Our review protocol was registered on PROSPERO (CRD42022379145). We searched the following databases: Central, MEDLINE, PubMed, Cochrane Library, Scopus, and LILACS between January 1947 and March 2023. Eligible studies included RCTs and observational studies evaluating strategies addressing weaning from mechanical ventilation among adults undergoing surgery. Additionally, we searched the Clinical Trials Registry Database (https://clinicaltrials.gov) for registered, unpublished, and ongoing studies evaluating weaning from mechanical ventilation in the operating room. We also searched bibliographies of included studies and review articles. Studies were restricted to English, French, Spanish, and Portuguese. For further information on the search strategy, please refer to Supplementary Appendix 1.

We included a study if it described at least one adult (>18 yr) patient being weaned from the ventilator in the operating room. Our primary outcomes were atelectasis measured by postoperative atelectasis and pulmonary aeration on computed tomography (CT) or lung ultrasound (LUS), and oxygenation through $Pa_{O_{2}}$, $Pa_{O_{2}}$/FiO_2_, estimated venous admixture, alveolar-to-arterial oxygen gradient, and supplemental oxygen use. Secondary outcomes included lung volume changes measured by functional residual capacity through inert gas rebreathing, end-expiratory lung volume with opto-electronic plethysmography, end-expiratory and total lung volume with electrical impedance tomography (EIT), and PPCs measured by the incidence of PPCs. We report our review based on PRISMA guidelines (Supplementary Appendix 2).^20^

### Mechanical ventilation intervention and control groups

Intervention groups included strategies examining facets of mechanical ventilation such as FiO_2_, PEEP, mode of ventilation, and recruitment manoeuvres. Control groups were defined as receiving either common or non-personalised care.

### Study selection

Two authors (MA and NS) independently reviewed retrieved abstracts and assessed eligibility using Covidence systematic review software (Veritas Health Innovation, Melbourne, VIC, Australia). A full-text review was conducted when either reviewer considered an abstract met inclusion criteria. Both reviewers agreed on full texts for inclusion, with an independent third reviewer (SMP) resolving disagreement.

### Data extraction

Data from included studies were independently extracted by MA and SMP. The following data were extracted: study and patient characteristics, mechanical ventilation settings, study interventions, perioperative ventilatory management including induction, intraoperative, and weaning phases, and outcomes. Two independent authors (MA and SMP) independently assessed the risk of bias at the outcome level with regards to randomisation, deviations from intended interventions, missing outcome data, and selection of the reported result using the Cochrane Risk of Bias tool for randomised studies (RoB2).^21^ A third review author (MCS) resolved any discrepancy that arose in the assessment of the process. Quality of evidence for each study was assessed by MA and MCS using the GRADE framework and are reported by intervention.^22^ At the time of registration, we considered performing a meta-analysis; however, this was not feasible because of the low number of eligible studies and heterogeneity of interventions and outcomes studied.

## Results

### Search results

**Fig 1:**
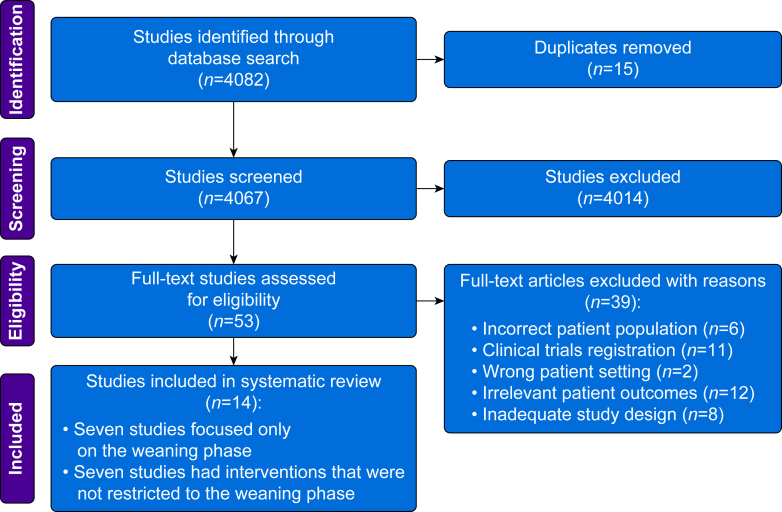
Identification of literature from search strategy based on inclusion and exclusion criteria.

Our search strategy identified 4082 citations that resulted in the inclusion of 14 studies based on screening eligibility (Fig. 1).

### Characteristics of included studies

Overall, 14 studies were included; seven studies examined the weaning phase only (Table 1) and the other seven studies addressed induction and maintenance in addition to weaning (Table 2).

**Table 1:** Interventions and physiological outcomes from studies that examined only the weaning period during mechanical ventilation, presented as Control *vs* Intervention 1 *vs* Intervention 2. ∗Values estimated from a figure. ABG, arterial blood gas; COPD, chronic obstructive pulmonary disease; CPAP, continuous positive airway pressure; CT, computed tomography; EIT, electrical impedance tomography; FiO_2_, fraction of inspired oxygen; LRM, lung recruitment manoeuvre; LUS, lung ultrasound; $Pa_{O_{2}}$, partial pressure of oxygen in arterial blood; $Pa_{O_{2}}$/FiO_2_, ratio of arterial oxygen partial pressure to fractional inspired oxygen; PEEP, positive end-expiratory pressure; PSV, pressure support ventilation; ZEEP, zero end-expiratory pressure.

Title of the study	Reference	Population	Weaning			Postoperative outcome and results	Observation
			Control	Intervention 1	Intervention 2		
The effect of increased FiO_2_ before tracheal extubation on postoperative atelectasis	Benoît and colleagues^23^	30 patients undergoing orthopaedic surgery under general anaesthesia.	FiO_2_ 1.0	FiO_2_ 1.0 + Recruitment manoeuvre	FiO_2_ 0.4 + Recruitment manoeuvre	1. Postoperative atelectasis (CT-measured): 8.3 (6.2)% *vs* 6.8 (3.4)% *vs* 2.6 (1.1)%, *P*<0.05 2. $Pa_{O_{2}}$ at PACU (ABG-measured): 11.3 *vs* 9.9 *vs* 12.7 kPa (*P*<0.05 when Intervention 2 *vs* Control)∗	Interventions performed 10 min before the presumed end of surgery.
Lung recruitment and positive airway pressure before extubation does not improve oxygenation in the post-anaesthesia care unit: a randomized clinical trial	Lumb and colleagues^14^	44 patients undergoing surgery with endotracheal intubation.	No LRM + PEEP ≤5 + no CPAP	LRM + PEEP 10 + CPAP 10 until extubation	–	1. Change (intraoperative – postoperative] in alveolar-to-arterial oxygen partial pressure difference (ABG-measured): 0.26 (0.87) *vs* 0.20 (0.89) kPa, *P*=0.82	Two ABGs drawn: intraoperatively and 1 h after extubation on FiO_2_ 0.4.
Pulmonary function after emergence on 100% oxygen in patients with chronic obstructive pulmonary disease: a randomized, controlled trial	Kleinsasser and colleagues^12^	53 patients undergoing carotid endarterectomy under general anaesthesia.	FiO_2_ 1.0	FiO_2_ 0.3	–	1. $Pa_{O_{2}}$ 5 min (ABG-measured): 16.7 (0.93) *vs* 8.1 (0.93) kPa 2. $Pa_{O_{2}}$ 15 min (ABG-measured): 8.1 (0.93) *vs* 9.1 (0.93) kPa, *P*<0.05 3. $Pa_{O_{2}}$ 60 min (ABG-measured): 8.3 (0.79) *vs* 8.9 (0.93) kPa, *P*<0.05	1. Only COPD patients 2. ABGs taken before induction and at 5, 15, and 60 min after extubation.
Positive end-expiratory pressure and postoperative atelectasis: a randomized controlled trial	Östberg and colleagues^10^	30 patients undergoing hernia or orthopaedic surgery under general anaesthesia.	ZEEP	PEEP 7 or 9	–	1. Postoperative atelectasis (CT-measured): 4.9 (3.0–12.7) *vs* 5.2 (2.4–14.3] cm^2^ – difference 0.3 cm^2^ (95% CI –1.5–2.0, *P*=0.854) 2. $Pa_{O_{2}}$/FiO_2_ 15–45 min after extubation (ABG-measured): 55.7 (46.5–77.6) *vs* 56.4 (41.1–73.8) kPa, *P*=0.961	CT performed approximately 30 min after extubation.
Effects of inspired oxygen concentration during emergence from general anesthesia on postoperative lung impedance changes evaluated by electrical impedance tomography: a randomised controlled trial	Park and colleagues^24^	71 patients undergoing elective laparoscopic colorectal surgery.	LRM + FiO_2_ 1.0	LRM + FiO_2_ 0.8	LRM + FiO_2_ 0.4	1. End-expiratory lung impedance ΔEELI/pre-EELI (EIT-measured): 46 (14)% *vs* 41 (14)% *vs* 37 (13)%, *P*=0.125 2. Total lung impedance reduction (EIT-measured): 49 (20)% *vs* 44 (17)% *vs* 40 (20)%, *P*=0.276	In PACU, all patients were given 5 L min^−1^ via a facial mask and the actual FI was approximately 0.4.
Pressure support versus spontaneous ventilation during anesthetic emergence – effect on postoperative atelectasis: a randomised controlled trial	Jeong and colleagues^25^	97 adult patients undergoing laparoscopic colectomy or robot-assisted prostatectomy.	Allow the patient to breathe and only help if necessary, with intermittent manual assistance	Driving pressure of 5 + PEEP 5 + backup ventilation 12 bpm	–	1. Incidence of atelectasis at PACU (LUS-measured): 57% *vs* 33% (RR 0.58; 95% CI 0.35–0.91, *P*=0.024) 2. $Pa_{O_{2}}$ at PACU (ABG-measured): 11.1 (1.7) *vs* 12.3 (3.5) kPa, *P*=0.034	–
Effects of an open lung extubation compared to a conventional extubation strategy on postoperative pulmonary complications after general anesthesia: a single-center pilot randomized controlled trial	Girard and colleagues^26^	69 patients at moderate to high risk of PPCs and undergoing intra-abdominal or non-thoracic surgery.	Dorsal decubitus + FiO_2_ 1.0 + manual bag ventilation	Semi-recumbent position + FiO_2_ 0.5 + PSV with unchanged PEEP	–	1. Difference between pulmonary aeration prior to emergence and PACU (LUS-measured): 0.3 (3.7) *vs* –1.6 (3.6), mean difference –1.9 (–3.7 to –0.1) 2. Oxygen supplementation during the first postoperative week (in % of hours): 58 (8–144) *vs* 12 (5–37), risk difference 9 (9–27) 3. Duration of supplemental O_2_ administration (in hours): 13 (2–26) *vs* 26 (22–28), median of difference 0 (–1 to 3)	Pilot study with multiple outcomes

**Table 2:** Interventions and physiological outcomes from studies that examined the intraoperative and/or maintenance phases in addition to the weaning phase during mechanical ventilation, presented as Control *vs*. Intervention 1 *vs*. Intervention 2 *vs*. Intervention 3. ∗Values estimated from a figure. CPAP, continuous positive airway pressure; CT, computed tomography; EIT, electrical impedance tomography; FiO_2_, fraction of inspired oxygen; FRC, functional residual capacity; LUS, lung ultrasound; $Pa_{O_{2}}$, partial pressure of oxygen in arterial blood; $Pa_{O_{2}}$/FiO_2_, ratio of arterial oxygen partial pressure to fractional inspired oxygen; PEEP, positive end-expiratory pressure; PPCs, postoperative pulmonary complications; PSV, pressure support ventilation; VCV, volume-controlled ventilation; ZEEP, zero end-expiratory pressure.

Title of the study	Reference	Population	Induction		Maintenance		Weaning				Postoperative outcome and results	Observation
			Control	Interventions 1 *vs* 2 *vs* 3	Control	Interventions 1 *vs* 2 *vs* 3	Control	Intervention 1	Intervention 2	Intervention 3		
Influence of perioperative oxygen fraction on pulmonary function after abdominal surgery: a randomized controlled trial	Staehr and colleagues^27^	35 patients undergoing laparotomy for ovarian cancer.	FiO_2_ 1.0	FiO_2_ 1.0	PEEP 5 + FiO_2_ 0.8	PEEP 5 + FiO_2_ 0.3	FiO_2_ 0.8 until extubation, then O_2_ 14 L min^−1^ and air 2 L min^−1^ on non-rebreathing facemask in PACU	FiO_2_ 0.3 until extubation, then O_2_ 2 L min^−1^ and air 14 L min^−1^ on non-rebreathing facemask in PACU	–	–	1. $Pa_{O_{2}}$/FiO_2_ 90 min after extubation (ABG-measured): 56.9 (45.9–66.9) *vs* 57.9 (39.9–69.9) kPa, *P*=0.66 2. FRC (inert gas-rebreathing method-measured): 1633 ml (1343–1948) *vs* 1615 ml (1375–2318), *P*=0.70	Intraoperative and weaning; FiO_2_ increased to 1.0 immediately before extubation.
Post-operative atelectasis – a randomised trial investigating a ventilatory strategy and low oxygen fraction during recovery	Edmark and colleagues^13^	59 patients undergoing orthopaedic surgery under general anaesthesia.	FiO_2_ 1.0 + CPAP 6, 7, or 8	FiO_2_ 1.0 + CPAP 6, 7, or 8	PEEP 6–8	PEEP 6–8	FiO_2_ 1.0	FiO_2_ 1.0	–	–	1. Atelectasis area (CT-measured): 6.8 (0–27.5) *vs* 5.5 (0–16.9) cm^2^, *P*=0.48	CPAP depending on weight during induction for all patients.
Preserved oxygenation in obese patients receiving protective ventilation during laparoscopic surgery: a randomized controlled study	Edmark and colleagues^28^	40 patients undergoing gastric bypass laparoscopic surgery.	FiO_2_ 1.0 + no CPAP	FiO_2_ 1.0 + CPAP 10 ***vs*** FiO_2_ 1.0 + CPAP 10	VCV + FiO_2_ 0.4 + PEEP 10	VCV + FiO_2_ 0.4 + PEEP 10 **vs.** VCV + FiO_2_ 0.4 + PEEP 10	FiO_2_ 1.0 + PEEP 10	FiO_2_ 1.0 + PEEP 10	FiO_2_ 0.31 + PEEP 10		1. Oxygenation (estimated venous admixture-measured): 14.2% *vs* 12.7% *vs* 8.1%	Induction, intraoperative, and weaning.
Individualised perioperative open-lung approach versus standard protective ventilation in abdominal surgery (iPROVE): a randomized controlled trial	Ferrando and colleagues^29^	965 patients undergoing abdominal surgery >2 h.	FiO_2_ 0.8	FiO_2_ 0.8 ***vs*** FiO_2_ 0.8 ***vs*** FiO_2_ 0.8 ***vs*** FiO_2_ 0.8	No LRM + PEEP 5	No LRM + PEEP 5 ***vs*** LRM + individual PEEP ***vs*** LRM + individual PEEP + individual CPAP	FiO_2_ 0.8 + PEEP 5 during weaning, then FiO_2_ 0.5 through Venturi face mask	FiO_2_ 0.8 + PEEP 5 during weaning, then FiO_2_ 0.5 through Venturi face mask + standard CPAP as rescue therapy	FiO_2_ 0.8 + individual PEEP during weaning, then FiO_2_ 0.5 through Venturi face mask + standard CPAP as rescue therapy	FiO_2_ 0.8 + individual PEEP during weaning, then FiO_2_ 0.5 through Venturi face mask + individual CPAP as rescue therapy	1. Number of patients with PPCs: 48% *vs* 43% (RR 0.90 [0.74–1.10]) *vs* 41% (RR 0.84 [0.69–1.03]) *vs* 39% (RR 0.80 [0.65–0.99])	Intraoperative and weaning, including rescue therapy.
Individual positive end-expiratory pressure settings optimize intraoperative mechanical ventilation and reduce postoperative atelectasis	Pereira and colleagues^11^	40 patients under general anaesthesia: 20 laparoscopic and 20 open abdominal.	FiO_2_ 1.0	FiO_2_ 1.0	PEEP 4	PEEP-EIT	PSV + FiO_2_ 0.5 + PEEP 4	PSV + FiO_2_ 0.5 + randomised PEEP	–	–	1. Percent of nonaerated lung tissue (CT-measured): 10.8 (7.1)% *vs* 6.2 (4.1)%, *P*=0.01	Intraoperative and weaning.
Specific anesthesia-induced lung volume changes from induction to emergence: a pilot study	Kostic and colleagues^30^	14 patients undergoing ENT surgery under general anaesthesia.	FiO_2_ 1.0	FiO_2_ 1.0	No LRM + ZEEP + FiO_2_ 0.4	LRM + PEEP 7 + FiO_2_ 0.4	No CPAP + FiO_2_ 1.0 for 5 min emergence	Pressure-limiting valve at 7 + FiO_2_ 0.4	–	–	1. End-expiratory lung volume when compared with baseline (opto-electronic plethysmography-measured): –0.6 *vs* +0.5 L, *P*<0.001∗	Intraoperative and weaning.
Perioperative high inspired oxygen fraction induces atelectasis in patients undergoing abdominal surgery: a randomized controlled trial	Park and colleagues^31^	172 patients older than 50 yr undergoing abdominal surgery under general anaesthesia.	FiO_2_ 1.0	FiO_2_ 0.7	FiO_2_ 0.6	FiO_2_ 0.35	FiO_2_ 1.0 during weaning, then 10 L min^−1^ via non-rebreathing for 15 min in PACU	FiO_2_ 0.7 during weaning, then 5 L min^−1^ via non-rebreathing for 15 min in PACU	–	–	1. LUS score: 7 (3–9) *vs* 3 (1–6), *P*<0.001 2. Significant atelectasis (LUS-measured): 39% *vs* 20% (RR 0.512 [95% CI 0.311–0.843, *P*=0.006])	Intraoperative and weaning.

A total of 1719 patients were included among the 14 studies. Patient characteristics and surgical procedures are outlined in Table 3. The primary outcome of atelectasis was examined including: postoperative atelectasis in five studies (*n*=388)^10^^,^^13^^,^^23^^,^^25^^,^^31^ and pulmonary aeration in two studies (*n*=109).^11^^,^^26^ Our second primary outcome of oxygenation was evaluated through $Pa_{O_{2}}$ in three studies (*n*=180),^12^^,^^23^^,^^25^ $Pa_{O_{2}}$/FiO_2_ in two studies (*n*=65),^10^^,^^27^ and in individual studies: alveolar-to-arterial oxygen pressure gradient (*n*=44),^14^ estimated venous admixture (*n*=40),^28^ LUS score (*n*=172),^31^ and oxygenation supplementation (*n*=69).^26^ A secondary physiologic outcome of lung volume changes was reported in individual studies including lung volume changes: functional residual capacity (*n*=35)^26^; end-expiratory and total lung impedance (*n*=71),^24^ end-expiratory lung volume (*n*=14)^30^; and secondary clinical outcome of PPCs was also reported in an individual study: incidence of PPCs (*n*=965).^29^

**Table 3:** Characteristics of patients who underwent various surgical procedures in included studies. Data presented as median (range), *n*/total N (%), or mean (sd). ASA, American Society of Anesthesiologists; ENT, ear, nose, and throat.

Characteristic	Control group (*n*=609)	Intervention group (*n*=1110)
Age (yr)	61.4 (21–89)	58.9 (28.5–85)
Sex, female	225/589 (38.2%)	420/1095 (38.4%)
BMI (kg m^−2^)	26.8 (3.7)	26.1 (4.3)
Height (cm)	166.4 (2.2)	166.1 (3.1)
Weight (kg)	77.3 (14.3)	76.1 (18.6)
ASA physical status (1/2/3)	84/287/135	316/390/261

Surgical intervention	No. of patients
Abdominal	1281
Gastric bypass	40
Hernia repair	29
Orthopaedic	90
Prostatectomy	97
Carotid endarterectomy in COPD	53
Laparotomy for ovarian cancer	35
ENT	14
Colorectal	71
Peripheral	9

### Studies that exclusively addressed the weaning phase

Seven studies that included 394 patients addressed the weaning phase exclusively, of which two studies examined a single intervention,^10^^,^^12^ and five examined multiple interventions (Table 1).^14^^,^^23^, ^24^, ^25^, ^26^ Control groups included varied strategies including zero end-expiratory pressure, intermittent manual assistance bag ventilation, and high FiO_2_.

Five studies examined multiple interventions. The combination of lower FiO_2_ with a recruitment manoeuvre was associated with less atelectasis [2.6 (1.1)% *vs* 8.3 (6.2)%] and improved $Pa_{O_{2}}$ (12.7 *vs* 11.3 kPa) compared with high FiO_2_ and no recruitment manoeuvre.^23^ Patients that received a combination of pressure support ventilation (PSV) and PEEP of 5 cm H_2_O demonstrated improved atelectasis (33% *vs* 57%) and oxygenation [12.3 (3.5) *vs* 11.1 (1.7) kPa].^25^ PSV with PEEP and a low FiO_2_ with a change in bed decubitus improved pulmonary aeration based on LUS scores and reduced the risk of oxygen supplementation (12% *vs* 58%).^26^ However, no difference was found in end-expiratory or total lung impedance among three different FiO_2_ concentrations with a lung recruitment manoeuvre.^24^ A PEEP <5 cm H_2_O compared with a PEEP of 10 cm H_2_O did not lead to a change in alveolar-to-arterial pressure difference.^14^

Two studies examined a single intervention. In patients with chronic obstructive pulmonary disease (COPD), an FiO_2_ of 0.3 compared with an FiO_2_ of 1.0 improved $Pa_{O_{2}}$ (8.1 [0.93] *vs* 16.7 [0.93] *vs* kPa) in PACU immediately after extubation).^12^ In another study, zero end-expiratory pressure compared with a PEEP of 7 or 9 cm H_2_O did not reduce the area of postoperative atelectasis or improve the $Pa_{O_{2}}$/FiO_2_ ratio.^10^

### Studies that addressed weaning in addition to induction or maintenance phase

Seven studies addressed the weaning phase in addition to the induction or intraoperative phases and included a total of 1325 patients (Table 2).^11^^,^^13^^,^^27^, ^28^, ^29^, ^30^, ^31^ Control groups included varied strategies: varied PEEP (ranging from 0 to 10 cm H_2_O); lung recruitment manoeuvres; high FiO_2_; and both support and controlled ventilation modes.

Low FiO_2_ was evaluated in four studies. Lower FiO_2_ during induction, maintenance, and weaning led to improved LUS scores and less atelectasis (20% *vs* 39%).^31^ A lower FiO_2_ during weaning also improved postoperative oxygenation (estimated venous admixture 8.1% *vs* 14.2%).^28^ However, the same group published another study where there was no change in atelectasis area when patients were weaned on 0.3 or 1.0 FiO_2_.^13^ High *vs* low FiO_2_ during maintenance and weaning also did not result in differences in oxygenation or functional residual capacity.^27^

An improvement in end-expiratory lung volume was found when patients received a combination of lung recruitment manoeuvres and PEEP during maintenance and CPAP with an FiO_2_ of 0.4 and with a pressure-limiting valve at 7 cm H_2_O throughout weaning compared with a group receiving FiO_2_ 1.0 and no CPAP for emergence (+0.5 L lung volume *vs* –0.6 L).^30^ When using PSV during the weaning phase, individualised PEEP compared with a PEEP of 4 cm H_2_O during maintenance and weaning phases showed a reduction in atelectasis on CT (6.2% nonaerated tissue *vs* 10.8%).^11^ In a large multicentre study, individualised PEEP intraoperative strategy with individualised postoperative CPAP suggested an association with reduced PPCs, an exploratory secondary outcome, compared with a standard low tidal volume intraoperative strategy with postoperative oxygen therapy (39% *vs* 48%).^29^

### Methodological quality of included studies

A summary of evidence by intervention for each outcome is outlined in Table 4. For the primary outcome, moderate- to high-quality studies suggest that weaning strategies including a combination of PSV, PEEP, and low FiO_2_ likely improve atelectasis; these studies were at low risk of bias for outcome ascertainment albeit with some concerns. The same interventions also demonstrated benefit in terms of oxygenation based on moderate- to high-quality evidence, although with some concerns of risk of bias in outcome detection. Low FiO_2_ alone improved atelectasis and oxygenation based on low to moderate quality of evidence with risk of bias, but did not demonstrate benefit on lung volumes based on moderate evidence with low risk of bias. For secondary outcomes of lung volumes, PSV with PEEP and low FiO_2_ strategies demonstrated improved lung volumes, albeit based on low-quality evidence and high risk of bias. An individualised ventilation strategy with PEEP reduced PPCs based on a moderate quality of evidence with low risk of bias in outcome ascertainment. See Supplementary Appendix 3A for the risk-of-bias summary and Supplementary Appendix 3B for the risk-of-bias graph, summarised by study, and Supplementary Appendix 4 for GRADE quality of evidence assessment.

**Table 4:** Summary of the evidence for each outcome studied in the intervention group compared with the control group with GRADE quality of evidence, risk of bias, and overall effect assessed. ∗Values estimated from a figure. A-a, alveolar-arterial oxygen pressure difference; ABG, arterial blood gas; CT, computed tomography; EIT, electrical impedance tomography; FiO_2_, fraction of inspired oxygen; FRC, functional residual capacity; LUS, lung ultrasound; PEEP, positive end-expiratory pressure; PPCs, postoperative pulmonary complications; PSV, pressure support ventilation; qLUSS, quantitative lung ultrasound score.

Intervention	Intervention group	Control group	Outcome measured (source; units)	GRADE	Risk of bias	Overall effect
*Outcome: atelectasis*						
PSV and PEEP	33% (RR 0.58; 95% CI 0.35–0.91)	57%	Incidence of atelectasis (LUS-measured; in %)^25^	High ⊕⊕⊕⊕	Low	Likely beneficial
PSV and PEEP and FiO_2_	1.6 (3.6) 6.2 (4.1)%	0.3 (3.7) 10.8 (7.1)%	Mean difference in pulmonary aeration PACU *vs* pre-emergence (qLUSS score)^26^ Percentage of nonaerated lung tissue (CT-measured; in %)^11^	Moderate ⊕⊕⊕	Some concerns	Likely beneficial
FiO_2_	6.8 (3.4)% *vs* 2.6 (1.1)% 5.5 (0–16.9) cm^2^ 3 (1–6) and 20% (RR 0.512; 95% CI 0.311–0.843)	8.3 (6.2%) 6.8 (0–27.5) cm^2^ 7 (3–9) and 39%	Postoperative atelectasis (CT-measured; in %)^23^ Atelectasis area (CT-measured; in cm^2^)^13^ LUS score and significant atelectasis (LUS-measured; score and %, respectively)^31^	Low ⊕⊕	High	Likely beneficial
PEEP	5.2 (2.4–14.3) cm^2^	4.9 (3.0–12.7) cm^2^	Postoperative atelectasis (CT-measured; in cm^2^)^10^	High ⊕⊕⊕⊕	Low	No effect
*Outcome: oxygenation*						
PSV and PEEP	12.3 (3.5) kPa	11.1 (1.7) kPa	$Pa_{O_{2}}$ at PACU (ABG-measured; in kPa) [24]	High ⊕⊕⊕⊕	Low	Likely beneficial
PSV and PEEP and FiO_2_	12% and 26 (22–28) h	58% and 13 (2–26) h	Percentage requiring oxygen supplementation during first postoperative week and duration of supplemental O_2_ administration in hours^26^	High ⊕⊕⊕⊕	Low	Likely beneficial
FiO_2_	9.9 *vs* 12.7 kPa∗ 8.9 (0.93) kPa 57.9 (39.9–69.9) kPa 12.7% *vs* 8.1%	11.3 kPa∗ 8.3 (0.79) kPa 56.9 (45.9–66.9) kPa 14.2%	$Pa_{O_{2}}$ at PACU (ABG-measured; in kPa)^23^ $Pa_{O_{2}}$ 60 min postoperative (ABG-measured; in kPa)^12^ $Pa_{O_{2}}$/FiO_2_ 90 min after extubation (ABG-measured; in kPa)^27^ Estimated venous admixture (ABG-measured, in %)^28^	Moderate ⊕⊕⊕	Some concerns	Likely beneficial
PEEP	0.20 (0.89) kPa 55.7 (46.5–77.6) kPa	0.26 (0.87) kPa 56.4 (41.1–73.8) kPa	A-a partial pressure difference (ABG-measured kPa)^14^ $Pa_{O_{2}}$/FiO_2_ 15–45 min after extubation (ABG-measured; in kPa)^10^	Moderate ⊕⊕⊕	Some concerns	No effect
Outcome: lung volumes						
PSV and PEEP and FiO_2_	+0.5 L∗	–0.6 L∗	End-expiratory lung volume (opto-electronic plethysmography-measured; in L)^30^	Low ⊕⊕	High	Likely beneficial
FiO_2_	41 (14)% *vs* 37 (13)% and 44 (17)% *vs* 40 (20)% 1633 ml (1343–1948)	46 (14)% and 49 (20)% 1615 ml (1375–2318)	End-expiratory and total lung impedance reduction (EIT-measured; in %)^24^ FRC (inert gas-rebreathing method-measured; in ml)^27^	Moderate ⊕⊕⊕	Low	No effect
Outcome: PPCs						
FiO_2_ and PEEP	43% (RR 0.90; 95% CI 0.74–1.10) *vs* 41% (RR 0.84; 95% CI 0.69–1.03) *vs* 39% (RR 0.80; 95% CI 0.65–0.99)	48%	Percentage of patients with PPCs^29^	Moderate ⊕⊕⊕	Low	Possibly beneficial

## Discussion

This systematic review on weaning from perioperative mechanical ventilation demonstrates that limited evidence guides this crucial component of clinical anaesthesia performed for nearly every patient undergoing surgery with general anaesthesia. Few studies have been conducted examining the weaning and emergence phases and their impact on postoperative pulmonary outcomes; only seven studies exclusively assessed the weaning phase, and seven other studies addressed the weaning phase in addition to the induction and maintenance phases. Of the strategies examined, PSV in combination with PEEP and low FiO_2_ consistently improved atelectasis, oxygenation, and lung volumes based on moderate to high quality of evidence and low risk of bias. Low FiO_2_ alone improved atelectasis and oxygenation based on low to moderate quality of evidence with risk of bias, but did not demonstrate benefit on lung volumes. A fixed-PEEP strategy was not associated with improvements in atelectasis or oxygenation based on moderate- to high-quality evidence albeit with some concerns of bias; however, an individualised PEEP strategy suggested potential reduced incidence of PPCs but requires further study.

Among the interventions studied, PSV in combination with PEEP and low FiO_2_ consistently demonstrated improvement in atelectasis, oxygenation, and lung volumes. These findings were in line with its wide use during weaning from mechanical ventilation in ICU.^32^, ^33^, ^34^ Specifically, compared with unassisted ventilation, PSV increases driving pressure during inspiration, causing tidal recruitment and promoting lung expansion, while simultaneously decreasing work of breathing.^32^^,^^35^ Moreover, the level of support can be titrated to match ventilatory and oxygenation requirements.^35^ Among the studies included in this review, PSV was used to maintain PEEP but the level was applied variably: in one study, PSV was used with no clear indication on how the level was set.^11^ Another study set the level to generate a volume that was similar to the tidal volume used prior to emergence and was maintained until the trachea was extubated.^26^ Finally, one study applied a PSV intervention initially setting the level at 5 cm H_2_O and gradually adjusting this level according to the resulting respiratory rate and tidal volume.^25^ Although PSV was set differently and used in various combinations with other strategies, it improved atelectasis,^11^^,^^25^^,^^26^ oxygenation,^25^^,^^26^ and lung volumes.^30^ We propose that applying a PSV strategy to maintain tidal volume^25^^,^^26^ could have a role during weaning from mechanical ventilation in the operating room. The combination of PEEP and low FiO_2_ with PSV increases end-expiratory lung volume and counteracts airway closure, which might reverse or prevent atelectasis in patients undergoing surgery.^36^

Evidence suggests that a higher FiO_2_ contributes to absorption atelectasis^37^ and surfactant impairment.^38^ Congruent with this, our review found that low FiO_2_ alone reduced atelectasis and improved oxygenation but might not improve lung volumes. This is because lung volume, more specifically end-expiratory lung volume, is modified by pressure support and specifically PEEP levels.^30^^,^^39^ Two reasons could explain why low FiO_2_ strategies improved atelectasis but not lung volumes. Firstly, we hypothesise that low FiO_2_ is not sufficient alone and needs to be combined with another intervention to see positive results as shown with the application of low FiO_2_ with PSV and PEEP (whereby PSV and PEEP would increase end-expiratory lung volumes). Secondly, EIT indirectly assesses changes in lung volumes by directly measuring changes in lung impedance, and therefore might not be as not sensitive as CT, which could explain why no significant differences were detected.^24^ The World Health Organization has previously issued a strong recommendation of an FiO_2_ of 0.8 intraoperatively and for 6 h postoperatively for prevention of surgical site infection.^40^ However, these guidelines have drawn criticism as a result of their generation based on a subgroup analysis of patients under general anaesthesia with tracheal intubation,^40^^,^^41^ evidence of increased mortality with liberal oxygen therapy in critically ill patients,^42^ and their lack of description of harm when observational evidence has suggested increased risk of pulmonary complications with hyperoxia.^43^ Updated meta-analyses have suggested that evidence favouring high FiO_2_ became weaker,^41^ but also that there was no definitive evidence of harm from high FiO_2_.^44^ Our study provides support for lower FiO_2_ in the weaning phases of mechanical ventilation in terms of reducing atelectasis and improving oxygenation.

Interventions that used a fixed or non-individualised PEEP strategy did not improve atelectasis or oxygenation. These PEEP strategies might not have adequately ameliorated lung collapse, especially as PEEP requirements vary vastly.^45^ Fixed PEEP strategies have been associated with inconclusive evidence of benefit.^17^^,^^18^^,^^39^ We found that strategies that incorporated individualised PEEP demonstrated improvements in atelectasis and oxygenation.^10^^,^^11^ The optimal PEEP strategy during weaning continues to be subject of debate. Inappropriately set PEEP levels have the potential to induce lung stress in non-dependent areas without effectively addressing atelectasis. Although one included trial examining an open-lung ventilation strategy incorporating individualised PEEP and postoperative CPAP among patients undergoing abdominal surgery suggested a reduction in PPCs, it was not powered for this secondary outcome and any effect should be considered hypothesis-generating. Further, differences in postoperative care could explain any possible effect as the treating team could not be blinded to the use of postoperative CPAP.^29^ Lending support to the positive direct effect hypothesis of an individualised PEEP strategy on PPCs, a recent trial of individualised ventilation comprising a recruitment manoeuvre, individualised PEEP and postoperative respiratory support demonstrated lower risk of severe PPCs among patients undergoing lung resection.^46^ Further research and definitive trials are therefore required to address which patients benefit from individualised PEEP strategies, and how these strategies need to be implemented in anaesthetic practice both during the intraoperative and emergence phases.

Prior research has demonstrated that alveolar recruitment manoeuvres improved lung mechanics and collapsed lung.^19^ Although no specific technique is currently recommended, bag-squeezing is discouraged and ventilator-driven alveolar recruitment manoeuvres should be performed instead.^19^ Recruitment manoeuvres should be applied using either the shortest effective time and lowest effective pressure or fewest number of breaths.^19^ In this review, recruitment manoeuvres combined with low FiO_2_ improved atelectasis and oxygenation,^23^ but not lung volumes,^24^ and with PEEP they did not improve oxygenation.^14^ Our findings do not support their clinical utility; recruitment manoeuvres were not consistently associated with improved physiologic outcomes, and are not safe across all scenarios. A recent RCT in patients with acute respiratory failure demonstrated harm in patients who received a recruitment manoeuvre.^47^

The results of studies that combined interventions during different perioperative phases are consistent with studies limited to the weaning phase. Specifically, combinations including PSV and low FiO_2_ were associated with improvements in oxygenation. Further, a mixed intervention that included PSV, low FiO_2_, and PEEP also improved lung volumes. These results imply that mechanical ventilation strategies during other phases of anaesthesia need to be combined with an intervention during the weaning phase to yield optimal outcomes. Resumption of active expiration without ventilatory support during emergence further decreased lung volumes^30^; therefore, a combination of techniques to recruit the lungs, reduce atelectasis, and restore diaphragm tone,^48^ with proper reversal of neuromuscular blocking agents, could minimise anaesthesia-induced physiologic changes to the respiratory system.

The strengths of this review include: an extensive literature search examining multiple databases including unpublished studies; inclusion of publications including intraoperative mechanical ventilation in addition to the weaning phase; a pre-registered protocol and analytic plan; and examination of the strength of evidence in addition to risk of bias. There are also several limitations. Firstly, this is an understudied subject and our review comprised only seven studies that addressed the weaning phase exclusively. As the seven other studies analysed included interventions during the induction and maintenance phases, it is possible that these led to significant heterogeneity in observed postoperative pulmonary outcomes. Secondly, our review identified lack of a standard weaning strategy. This lack of a consistent control group limited analyses, as potential facets of mechanical ventilation differed in both intervention and control groups. Thirdly, the limited research to date comprises heterogeneous patient and surgical populations. Patients studied included those with COPD who have compromised pulmonary function; those undergoing abdominal and chest surgery with greater postoperative respiratory derangements; and those undergoing laparoscopic surgery which is associated with marked cardiorespiratory impairment. Therefore, differences in outcomes could be influenced by confounding preoperative and intraoperative factors. Fourthly, outcome definitions were inconsistent, such as studies of oxygenation including $Pa_{O_{2}}$, $Pa_{O_{2}}$/FiO_2_, and estimated venous admixture. Similarly, studies evaluating atelectasis used both CT, the gold-standard, and LUS, which has limitations in inter- and intra-rater reliability. Fifthly, interventions studied varied from a single approach to multiple strategies in numerous combinations, and this limited the assessments of benefit of each individual intervention. Finally, contemporary definitions of PPC do not incorporate quantitative assessments of atelectasis.^49^ Consequently, a reduction in atelectasis as reported among included studies might not inherently correspond to reductions in PPC. Nonetheless, such quantitative assessments are useful as atelectasis is a risk factor for further lung injury and reduction in atelectasis has potential to improve PPC.^5^^,^^8^

Given the paucity of studies on this topic, further research is needed to elucidate physiological mechanisms of specific weaning strategies, and their clinical implications. The potential for improved patient outcomes demonstrated in our review emphasises the importance of developing interventions that target this phase of anaesthesia. RCTs focused on developing individualised mechanical ventilation strategies with focus on the weaning phase are urgently needed. In the interim, based on the limited evidence reviewed here, we suggest that clinicians consider weaning patients from mechanical ventilation after surgery with a combination of PSV, individualised PEEP, and low FiO_2_, pending more definitive studies.

### Conclusions

This systematic review revealed limited data to guide weaning from intraoperative mechanical ventilation. The lack of a standard approach to weaning and limited evidence guiding this phase of anaesthesia result in considerable discrepancies in care. Based on low-quality evidence from randomised trials of modest sample sizes, the combination of PSV, individualised PEEP, and low FiO_2_ during weaning from mechanical ventilation is potentially associated with diminished atelectasis and enhanced oxygenation.

## Authors’ contributions

Conceptualized the project: MA, SMP, MG, AS, MCS

Evaluated all studies: MA, NS

Analysed the data: MA, SMP, MCS

Prepared the manuscript and created all tables, figures, and supplementary materials: MA, SMP

Contributed to editing the manuscript and approved the final version: all authors

## Declarations of interest

MA was supported by the Keenan Research Summer Student Award. AS and MCS are supported by Merit Awards from the Department of Anesthesiology and Pain Medicine at the University of Toronto. MG is a consultant for GE HealthCare's ultrasound point of care group. None of the funding is related to the conduct of this work. The remaining authors have no conflicts of interest.
