# Supplementary material for: Weaning from mechanical ventilation in the operating room: a systematic review
Source: Br J Anaesth. 2024 May 29;133(2):424–36. doi: 10.1016/j.bja.2024.03.043 (PMC11282496; doi:10.1016/j.bja.2024.03.043)
Supplement: Multimedia component 1 [file mmc1.docx]

Weaning from Mechanical Ventilation in the Operating Room - A Systematic Review

Megan Abbott*, Sérgio M Pereira*, Noah Sanders, Martin Girard, Ashwin Sankar°, Michael C Sklar°

*Authors contributed equally to the manuscript

°Sharing of senior authorship

SUPPLEMENTAL DIGITAL CONTENT

All Ovid Medline <1946 - present>

1 exp Respiration, Artificial/ (86687)

2 respiration.af. (232341)

3 (patient adj5 ventilate*).ti,ab,kf. (1222)

4 (patient* adj5 ventilator).ti,ab,kf. (5348)

5 (Pulmonary adj3 Ventilation).af. (16908)

6 respiratory insufficiency/th (13279)

7 exp ventilators, mechanical/ (10002)

8 (ventilator or ventilation).ti,ab,kf. (156995)

9 or/1-8 (355283)

10 exp airway extubation/ (2188)

11 (extubate: or extubation*).ti,ab,kf. (15809)

12 exp Ventilator Weaning/ (4314)

13 ventilator weaning.af. [all fields] (4621)

14 weaning, ventilator.af. (11)

15 (weaning adj3 ventilation).ti,ab,kf. [title abstract and author-supplied keyword] (2002)

16 (weaning adj3 postoperative).ti,ab,kf. (77)

17 (weaning adj3 pulmonary).ti,ab,kf. (79)

18 Post-operative period.mp. (4537)

19 exp Anesthesia Recovery Period/ (5483)

20 emergence.ti,ab,kf. (129980)

21 ((recovery or emergence) adj5 (anesthesia or Aenesthesia)).ti,ab,kf. (4455)

22 10 or 11 or 12 or 13 or 14 or 15 or 16 or 17 or 18 or 19 or 20 or 21 (160129)

23 exp Positive-Pressure Respiration/ (28463)

24 PEEP.af. (6472)

25 Electrical Impedance Tomography.af. (1991)

26 P F ratio.tw. (390)

27 fractional inspired oxygen.mp. (391)

28 23 or 24 or 25 (32829)

29 9 and 22 and 28 (1753)

30 exp Postoperative Complications/ (597776)

31 exp Oximetry/ (16394)

32 P F ratio.tw. (390)

33 fractional inspired oxygen.mp. (391)

34 PaO2.mp. (13815)

35 FiO2.mp. (9098)

36 postoperative pulmonary function.mp. (480)

37 exp Pulmonary Atelectasis/ (6950)

38 atelectasis.af. (11925)

39 30 or 31 or 32 or 33 or 34 or 35 or 36 or 37 or 38 (641090)

40 29 and 39 (414)

Keywords used for search strategy
