# Supplementary material for: Weaning from mechanical ventilation in the operating room: a systematic review
Source: Br J Anaesth. 2024 May 29;133(2):424–36. doi: 10.1016/j.bja.2024.03.043 (PMC11282496; doi:10.1016/j.bja.2024.03.043)
Supplement: Multimedia component 3 [file mmc3.docx]

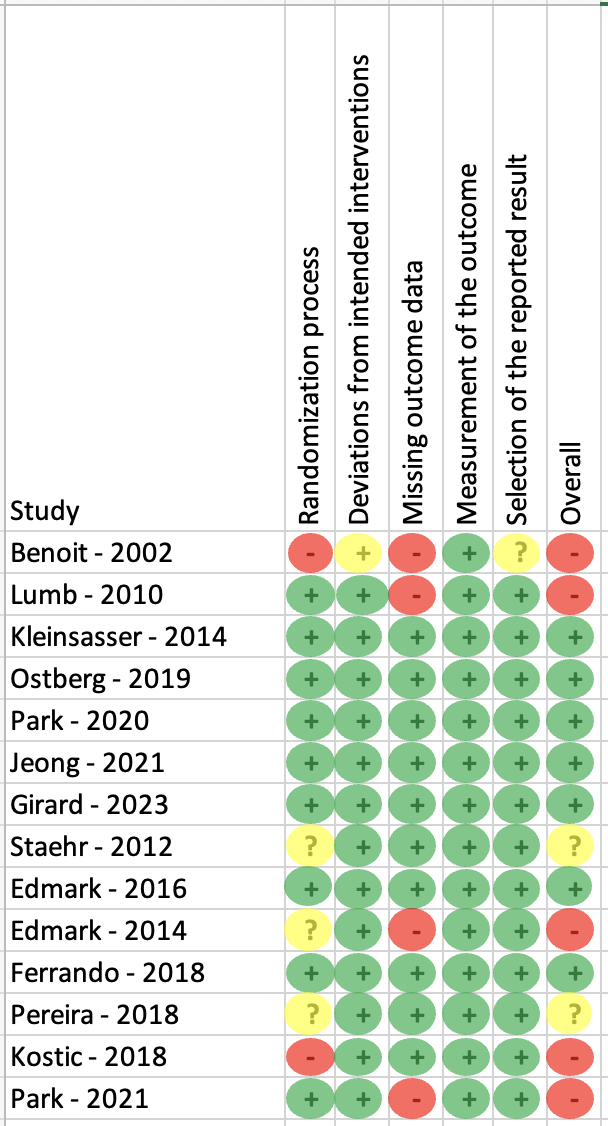

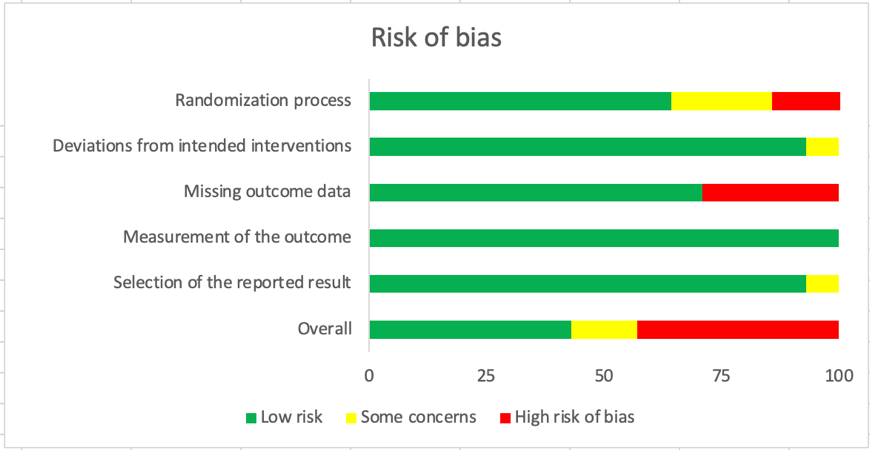


**A**

**B**

**Appendix 2A:** Overview of individual randomized-control trial study risk of bias assessment. Green circle represents a low risk of bias, yellow circle with a question mark there were some concerns of bias, and the red circle indicates high risk of bias.

**Appendix 2B:** Overview of risk of bias graph.
