# Supplementary material for: Weaning from mechanical ventilation in the operating room: a systematic review
Source: Br J Anaesth. 2024 May 29;133(2):424–36. doi: 10.1016/j.bja.2024.03.043 (PMC11282496; doi:10.1016/j.bja.2024.03.043)
Supplement: Multimedia component 4 [file mmc4.docx]

**Appendix 3:** Summary of GRADE quality of evidence assessment for individual studies in this systematic review and overall quality of evidence designation.

| **Study** | **Study Design** | **Inconsistency of Results** | **Indirectness of Evidence** | **Imprecision** | **Publication Bias** | **Quality of Evidence for Individual Study (n=1)** | **Overall Quality of Evidence for All Studies (n=15)** |
| --- | --- | --- | --- | --- | --- | --- | --- |
| Benoit 2002 | Randomized trial | Very serious risk of bias | Not serious | Not serious | Undetected | Low  ⊕⊕ | Low  ⊕⊕ |
| Lumb 2010 | Randomized trial | Serious risk of bias | Not serious | Not serious | Undetected | Moderate  ⊕⊕⊕ | Low  ⊕⊕ |
| Kleinsasser 2014 | Randomized trial | No serious risk of bias | Serious | Not serious | Undetected | Moderate  ⊕⊕⊕ | Low  ⊕⊕ |
| Ostberg 2019 | Randomized trial | No serious risk of bias | Not serious | Not serious | Undetected | High  ⊕⊕⊕⊕ | Low  ⊕⊕ |
| Park 2020 | Randomized trial | No serious risk of bias | Not serious | Not serious | Undetected | Moderate  ⊕⊕⊕ | Low  ⊕⊕ |
| Jeong 2021 | Randomized trial | No serious risk of bias | Not serious | Not serious | Undetected | High  ⊕⊕⊕⊕ | Low  ⊕⊕ |
| Girard 2023 | Randomized trial | No serious risk of bias | Not serious | Not serious | Undetected | High  ⊕⊕⊕⊕ | Low  ⊕⊕ |
| Staehr 2012 | Randomized trial | No serious risk of bias | Serious | Not serious | Undetected | Moderate  ⊕⊕⊕ | Low  ⊕⊕ |
| Edmark 2016 | Randomized trial | No serious risk of bias | Serious | Serious | Undetected | Low  ⊕⊕ | Low  ⊕⊕ |
| Edmark 2014 | Randomized trial | Serious risk of bias | Serious | Not serious | Undetected | Moderate  ⊕⊕⊕ | Low  ⊕⊕ |
| Ferrando 2018 | Randomized trial | No serious risk of bias | Serious | Not serious | Undetected | Moderate  ⊕⊕⊕ | Low  ⊕⊕ |
| Pereira 2018 | Randomized trial | No serious risk of bias | Serious | Not serious | Undetected | Moderate  ⊕⊕⊕ | Low  ⊕⊕ |
| Kostic 2018 | Randomized trial | Serious risk of bias | Serious | Not serious | Undetected | Low  ⊕⊕ | Low  ⊕⊕ |
| Park 2021 | Randomized trial | Serious risk of bias | Serious | Not serious | Undetected | Low  ⊕⊕ | Low  ⊕⊕ |
